# Supplementary material for: Parkinson’s Disease-vulnerable and -resilient dopamine neurons display opposite responses to excitatory input
Source: bioRxiv. 2025 Jun 7:2025.06.03.657460. Preprint. [Version 1] doi: 10.1101/2025.06.03.657460 (PMC12157410; doi:10.1101/2025.06.03.657460)
Supplement: Supplement 1 [file NIHPP2025.06.03.657460v1-supplement-1.pdf]

bioRxiv preprint doi: https://doi.org/10.1101/2025.06.03.657460; this version posted June 7, 2025. The copyright holder for this preprint (which was not certified by peer review) is the author/funder, who has granted bioRxiv a license to display the preprint in perpetuity. It is made available under aCC-BY 4.0 International license.

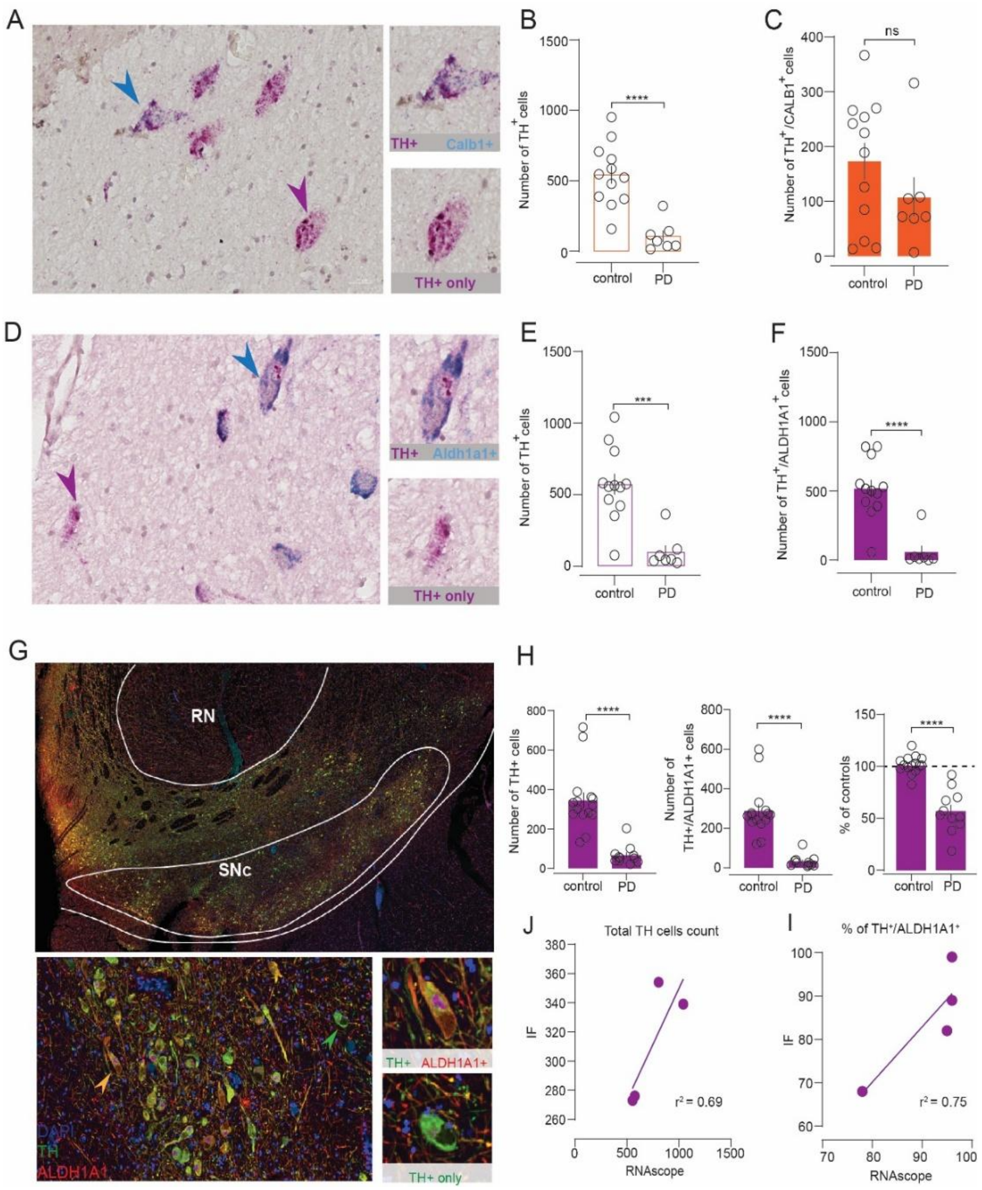

Supplemental figure 1 (related to figure 1). Quantification of *CALB1* and *ALDH1A1* populations in SNc from same cases as described for *ANXA1*. A) Example images showing SNc DA neurons expressing *TH* alone (purple arrow) or both *TH* and *CALB1* (blue arrow). Scale: 50 µm (top), 10 µm (bottom). **B)** *TH*+ neuron counts were significantly reduced in PD cases (n=7) compared to controls. **C)** *TH+*/*CALB1*+ neuron counts were unchanged between groups. **D)** Same as A), showing *TH* and *ALDH1A1* labeling. **E)** Same analysis as **B)** for *ALDH1A1*. **F)** $TH^{+}/ALDH1A1^{+}$ counts are significantly reduced in PD. **G)** Representative immunofluorescence (IF) images at low and high magnification showing *DAPI* (blue), *TH* (green), and *ALDH1A1* (red) in SNc from a control case. **H)** Quantification of IF showing significant decrease in TH+, $TH^{+}/ALDH1A1^{+}$, and % *TH+/ALDH1A1+* showing de-enrichment in PD (n=11) relative to control (n=15). **I)** A subset of cases (n=4) were processed by both IF and RNAscope, showing correlated TH+ counts and **J)** fraction of *TH*+ cells expressing *ALDH1A1*. Unpaired t-test or Mann-Whitney, ***p<0.001, ****p<0.0001.

bioRxiv preprint doi: https://doi.org/10.1101/2025.06.03.657460; this version posted June 7, 2025. The copyright holder for this preprint (which was not certified by peer review) is the author/funder, who has granted bioRxiv a license to display the preprint in perpetuity. It is made available under aCC-BY 4.0 International license.

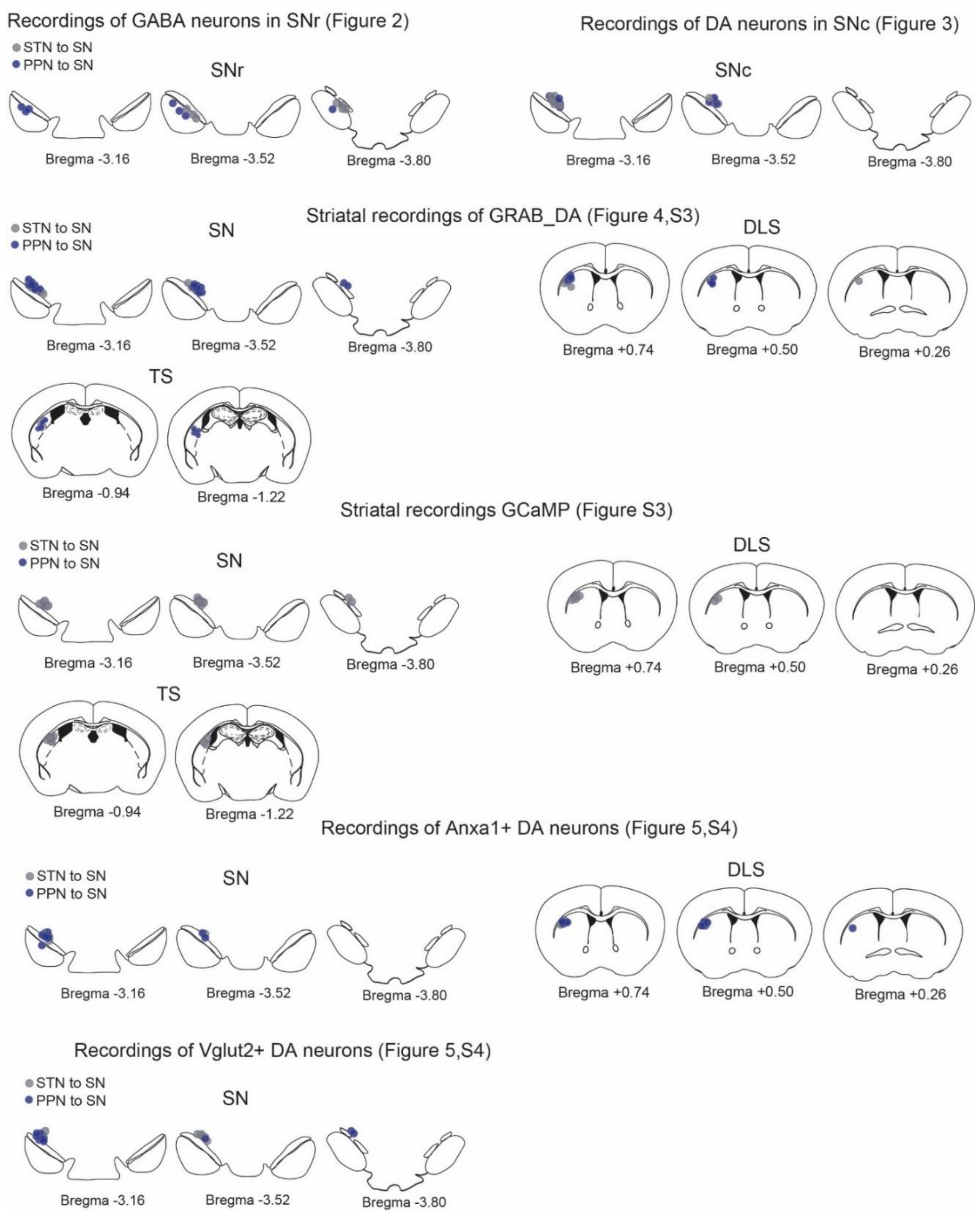

Supplemental Figure 2 (related to Figures 2-5, S3-S4). Maps showing fiber placement for each animal included in Figures 2-5 and S3-S4 in SN, DLS, or TS. All placements depicted on the left hemisphere here for ease of comparison. Actual implants were counterbalanced between the left vs. right hemispheres across mice.

bioRxiv preprint doi: https://doi.org/10.1101/2025.06.03.657460; this version posted June 7, 2025. The copyright holder for this preprint (which was not certified by peer review) is the author/funder, who has granted bioRxiv a license to display the preprint in perpetuity. It is made available under aCC-BY 4.0 International license.

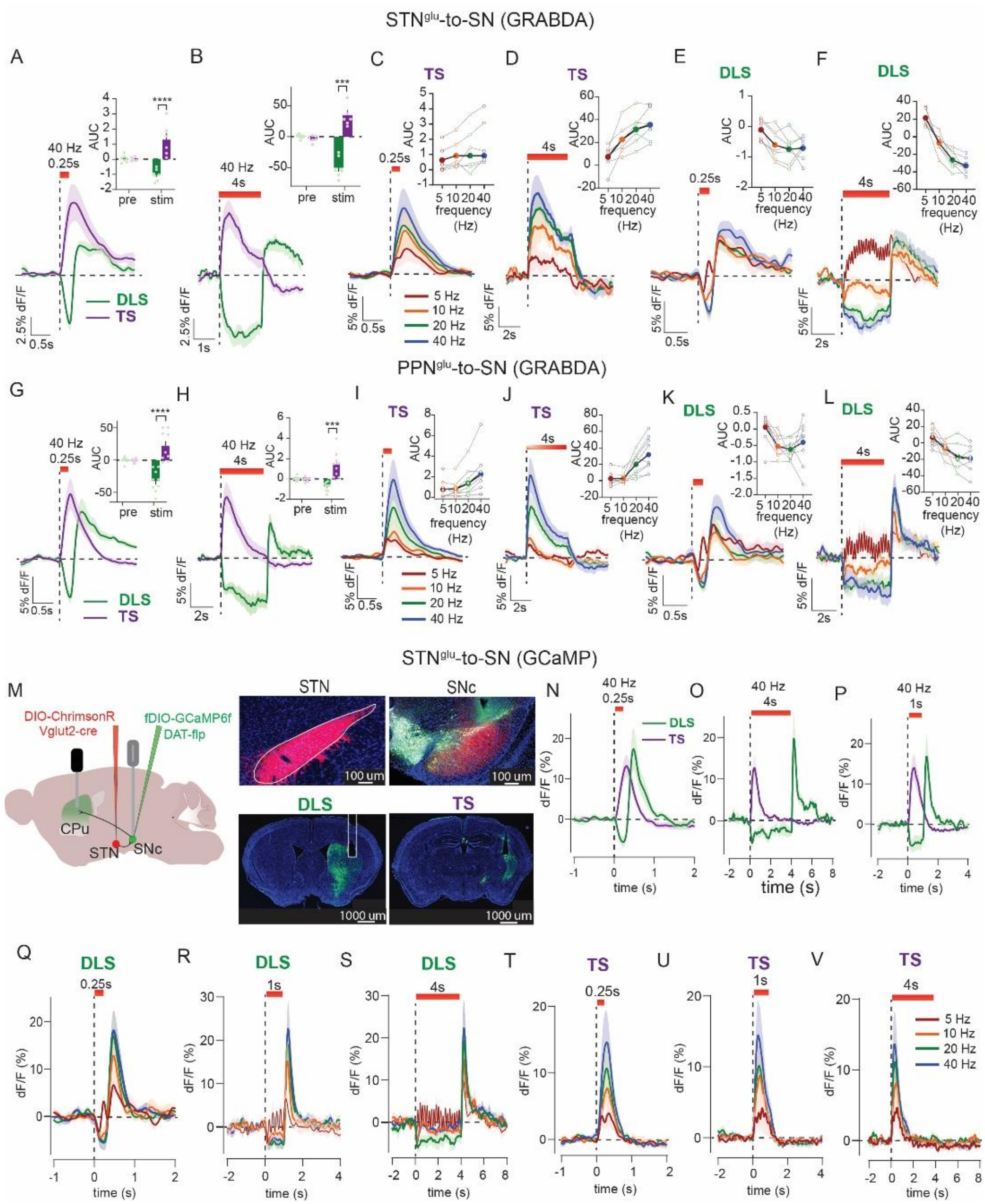

Supplemental figure 3 (related to figure 4). Comparing DA responses to STN and PPN stimulation across additional parameters. A) Optogenetic stimulation of STN inputs for 0.25 s or **B**) 4 s evoked DA release measured by GRAB$_{DA}$ in TS (n=6) but led to suppression of DA in DLS (n=7). **C-F)** Varying frequency of STN stimulation with 0.25 s or 4 s trains produced frequency-dependent increases in TS or decreases in DLS. **G-L)** Same as **A-F)** but with stimulation of PPN inputs (TS and DLS: n=9). **I–L)** PPN stimulation produced a frequency-dependent increase in TS, and inhibition in DLS. **M)** Experimental strategy to stimulate STN inputs to SN and record the activity of SNc DA terminals in TS and DLS with GCaMP6f; histology showing ChrimsonR:TdTomato expression in STN neurons and SN terminals (red), plus GCaMP expression in SNc DA neurons and their terminals in DLS or TS, sections counter-stained with DAPI (blue) and in SNc with TH (white); scale 1 mm. **N-V)** Stimulation of STN inputs to SN and recordings of activity in DA terminals in TS (n=9) or DLS (n=9). Sidak's post hoc, ***p<0.001, ****p<0.0001.

bioRxiv preprint doi: https://doi.org/10.1101/2025.06.03.657460; this version posted June 7, 2025. The copyright holder for this preprint (which was not certified by peer review) is the author/funder, who has granted bioRxiv a license to display the preprint in perpetuity. It is made available under aCC-BY 4.0 International license.

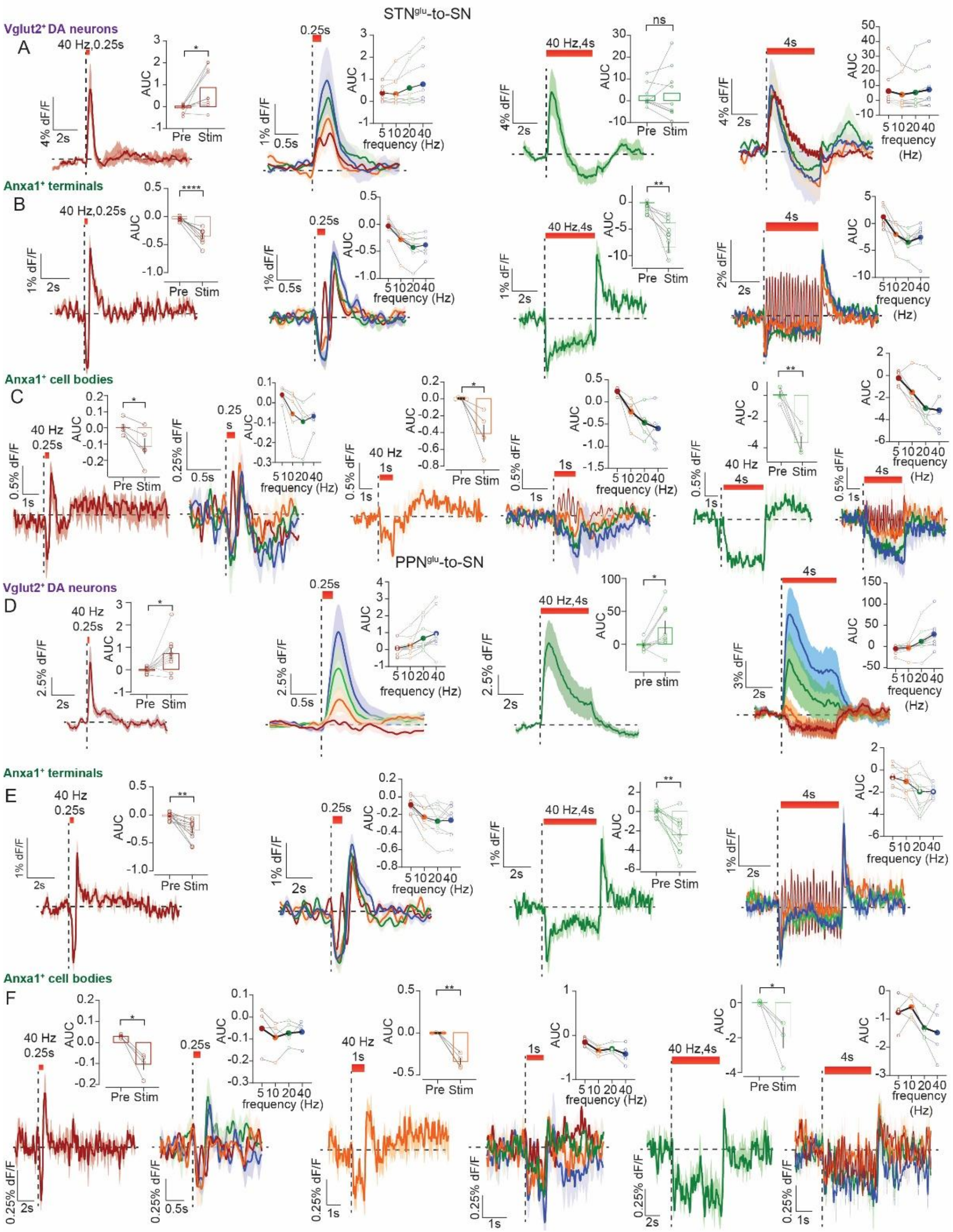

Supplemental Figure 4 (related to Figure 5). Comparing DA subtype responses to STN and PPN stimulation across additional parameters. **A)** Stimulation of STN inputs to SN with GCaMP8f recordings from Vglut2+ DA neurons (n=10). **B)** Stimulation of STN inputs to SN plus GCaMP8f recordings from Anxa1+ SN terminals in DLS (n=8). **C)** Stimulation of STN inputs to SN plus GCaMP8f recordings from Anxa1+ soma in SNc (n=5). **D)** Stimulation of PPN inputs to SN with GCaMP8f recordings from Vglut2+ DA neurons (n=10). **E)** Stimulation of PPN inputs to SN plus GCaMP8f recordings from Anxa1+ SN terminals in DLS (n=10). **F)** Stimulation of PPN inputs to SN plus GCaMP8f recordings from Anxa1+ soma in SNc (n=4). Paired t-test or Wilcoxon, *p<0.05, **p<0.01, ****p<0.0001

bioRxiv preprint doi: https://doi.org/10.1101/2025.06.03.657460; this version posted June 7, 2025. The copyright holder for this preprint (which was not certified by peer review) is the author/funder, who has granted bioRxiv a license to display the preprint in perpetuity. It is made available under aCC-BY 4.0 International license.

Case #	Sex	Age (yrs)	PMI (h)	Plate	Diagnosis	Source	Assessed for
BEB18119A032	M	72	18	3	control	NIH	mRNA
120927-22F	M	72	17	4	control	NIH	mRNA
BEB18084A001	M	67	26	5	control	NIH	mRNA
Hct16HDMA026	M	65	22	5	control	NIH	mRNA
118467-22F	M	71	13	5	control	NIH	mRNA and protein
118375-22F	M	52	12	5	control	CNDR	mRNA
118280-23F	F	56	21	5	control	CNDR	mRNA
BEB1907A024	M	93	17	6	control	CNDR	mRNA
BEB19019A014	M	67	12	6	control	CNDR	mRNA
120806-22F	M	54	21	6	control	CNDR	mRNA and protein
122016-25F	M	66	11	7	control	CNDR	mRNA and protein
118089-22F	M	59	17	7	control	CNDR	mRNA and protein
C085	F	81	22	3	control	PUKBB	protein
PDC034	M	90	N/G	4	control	PUKBB	protein
C083	F	87	24	4	control	PUKBB	protein
PDC056	F	70	N/G	4	control	PUKBB	protein
PDC053	F	89	N/G	5	control	PUKBB	protein
C028	F	60	13	6	control	PUKBB	protein
C066	M	84	23	7	control	PUKBB	protein
PDC041	M	66	N/G	7	control	PUKBB	protein
PDC019	F	74	N/G	7	control	PUKBB	protein
PDC032	F	91	N/G	7	control	PUKBB	protein
C037	M	84	5	8	control	PUKBB	protein
106586-1F	M	65	14	5	PD	CNDR	mRNA
104020-24F	M	76	15	5	PD	CNDR	mRNA
103780-39F	M	77	4	6	PD	CNDR	mRNA
112655-36F	M	85	13	4	PDD	CNDR	mRNA
100699-27F	M	79	12	5	PDD	CNDR	mRNA
106669-26F	M	86	16	5	PDD	CNDR	mRNA
115580-26F	M	68	4	6	PDD	CNDR	mRNA
105236-53F	M	66	4	6	PDD	CNDR	mRNA
112876-25F	M	76	5	6	PDD	CNDR	mRNA
PD154	F	76	N/G	3	PD	PUKBB	protein
PD427	M	79	N/G	4	PD	PUKBB	protein
PD069	M	82	N/G	5	PD	PUKBB	protein
PD110	M	72	N/G	5	PD	PUKBB	protein
PD137	F	88	N/G	5	PD	PUKBB	protein
PD506	M	67	N/G	5	PD	PUKBB	protein
PD054	M	75	N/G	6	PD	PUKBB	protein
PD141	F	75	N/G	6	PD	PUKBB	protein
PD062	M	62	N/G	7	PD	PUKBB	protein
PD161	F	72	N/G	7	PD	PUKBB	protein
PD199	F	83	N/G	8	PD	PUKBB	protein

Supplemental Table 1. Demographic and other data for cases assessed for mRNA, protein, or both. Level is based on the human brain atlas published by Coulomb et al. 2021 Front Neuroanat. Abbreviations: PD, Parkinson's disease; PDD, Parkinson's disease with dementia; NIH, National Institutes of Health NeuroBioBank; CDNR, Center for Neurodegenerative Disease Research at the University of Pennsylvania; PUKBB Parkinson's United Kingdom Brain Bank; N/G, not given.

bioRxiv preprint doi: https://doi.org/10.1101/2025.06.03.657460; this version posted June 7, 2025. The copyright holder for this preprint (which was not certified by peer review) is the author/funder, who has granted bioRxiv a license to display the preprint in perpetuity. It is made available under aCC-BY 4.0 International license.

Diagnosis	Case #	Plate	*TH+* Cell count	*TH+/CALB1+* Cell count	*TH+/CALB1-* Cell count	*CALB1+* %	*CALB1+* % of mean control
Control	BEB18119A032	3	330	1	329	0.3	3.3
	120927-22F	4	158	41	117	25.9	285.8
	BEB18084A001	5	654	77	577	11.8	129.7
	Hct16HDMA026	5	388	0	388	0.0	0.0
	118467-22F	5	813	150	663	18.5	203.2
	118375-22F	5	952	141	811	14.8	163.1
	118280-23F	5	523	29	494	5.5	61.1
	BEB1807A024	6	372	36	336	9.7	106.6
	BEB19019A014	6	585	50	535	8.5	94.1
	120806-22F	6	708	58	650	8.2	90.2
	122016-25F	7	546	28	518	5.1	56.5
	118089	7	478	3	475	0.6	6.9
PD	106586-1F	5	37	18	19	48.6	535.8
	104020-24F	5	39	27	12	69.2	762.5
	103780-39F	6	14	3	11	21.4	236.0
PDD	112655-36F	4	71	16	55	22.5	248.2
	100699-27F	5	143	2	141	1.4	15.4
	106669-26F	5	322	90	232	28.0	307.8
	115580-26F*	6	1	0	1	n/a	n/a
	105236-53F*	6	0	0	0	n/a	n/a
	112876-25F	6	117	0	117	0.0	0.0
	Mean (control)	5.3	542.3	51.2	491.1	9.1	100.0
	Mean (PD & PDD)	5.3	82.7	17.3	65.3	27.3	300.8
	SEM (control)	0.3	63.5	14.4	52.8	2.3	24.9
	SEM (PD & PDD)	0.3	40.0	11.9	31.6	9.4	103.2

Diagnosis	Case #	Plate	*TH+* Cell count	*TH+/ALDH1A1+* Cell count	*TH+/ALDH1A1-* Cell count	*ALDH1A1+* %	*ALDH1A1+* % of mean control
Control	BEB18119A032	3	423	422	1	99.8	111.1
	120927-22F	4	78	57	21	73.1	81.4
	BEB18084A001	5	884	822	62	93.0	103.5
	Hct16HDMA026	5	354	350	4	98.9	110.1
	118467-22F	5	805	767	38	95.3	106.1
	118375-22F	5	1043	820	223	78.6	87.5
	118280-23F	5	583	483	100	82.8	92.2
	BEB1807A024	6	467	391	76	83.7	93.2
	BEB19019A014	6	565	516	49	91.3	101.7
	120806-22F	6	558	500	58	89.6	99.8
	122016-25F	7	553	530	23	95.8	106.7
	118089	7	575	551	24	95.8	106.7
PD	106586-1F	5	39	12	27	30.8	34.3
	104020-24F	5	45	10	35	22.2	24.7
	103780-39F	6	26	9	17	34.6	38.5
PDD	112655-36F	4	74	21	53	28.4	31.6
	100699-27F	5	120	0	120	0.0	0.0
	106669-26F	5	364	328	36	90.1	100.3
	115580-26F*	6	3	3	0	n/a	n/a
	105236-53F*	6	0	0	0	n/a	n/a
	112876-25F	6	49	24	25	49.0	54.5
	Mean (control)	5.3	574.0	517.4	56.6	89.8	100.0
	Mean (PD & PDD)	5.3	80.0	45.2	34.8	36.4	40.6
	SEM (control)	0.3	72.8	62.4	17.3	2.4	2.7
	SEM (PD & PDD)	0.3	45.1	45.1	13.3	10.5	11.7

Diagnosis	Case #	Plate	*TH+* Cell count	*TH+/ANXA1+* Cell count	*TH+/ANXA1-* Cell count	*ANXA1+* %	*ANXA1+* % of mean control
Control	BEB18119A032	3	432	62	370	14.4	170.7
	120927-22F	4	133	10	123	7.5	89.4
	BEB18084A001	5	868	51	817	5.9	69.9
	Hct16HDMA026	5	368	40	328	10.9	129.2
	118467-22F	5	887	57	830	6.4	76.4
	118375-22F	5	1068	6	1062	0.6	6.7
	118280-23F	5	623	51	572	8.2	97.3
	BEB1807A024	6	410	12	398	2.9	34.8
	BEB19019A014	6	519	46	473	8.9	105.4
	120806-22F	6	788	53	735	6.7	80.0
	122016-25F	7	608	128	480	21.1	250.3
	118089	7	558	42	516	7.5	89.5
PD	106586-1F	5	30	1	29	3.3	39.6
	104020-24F	5	45	0	45	0.0	0.0
	103780-39F	6	29	0	29	0.0	0.0
PDD	112655-36F	4	79	0	79	0.0	0.0
	100699-27F	5	150	0	150	0.0	0.0
	106669-26F	5	335	9	326	2.7	31.9
	115580-26F*	6	0	0	0	n/a	n/a
	105236-53F*	6	0	0	0	n/a	n/a
	112876-25F	6	40	0	40	0.0	0.0
	Mean (control)	5.3	605.2	46.5	558.7	8.4	100.0
	Mean (PD & PDD)	5.3	78.7	1.1	77.6	0.9	10.2
	SEM (control)	0.3	75.5	9.3	75.1	1.5	18.2
	SEM (PD & PDD)	0.3	42.2	1.3	41.0	0.6	6.7

Supplemental Table 2. *TH*, *CALB1*, *ALDH1A1*, and *ANXA1* neuron counts in SNc of control and age-matched PD cases with RNAscope labeling. *Cases excluded from analyses because the total number of *TH*+ cells counted was too low.

bioRxiv preprint doi: https://doi.org/10.1101/2025.06.03.657460; this version posted June 7, 2025. The copyright holder for this preprint (which was not certified by peer review) is the author/funder, who has granted bioRxiv a license to display the preprint in perpetuity. It is made available under aCC-BY 4.0 International license.

Diagnosis	Case #	Plate	*TH+* Cell count	*TH+/ALDH1A1+* Cell count	*TH+/ALDH1A1-* Cell count	*ALDH1A1+* %	*ALDH1A1+* % of mean control
Control	C085	3	132	120	12	90.9	110.3
	PDC034	4	282	223	59	79.1	96.0
	C083*	4	302	263	39	87.1	105.7
	PDC056	4	332	269	63	81.0	98.3
	PDC053	5	156	130	26	83.3	101.1
	C028	6	362	277	85	76.5	92.9
	C066	7	301	190	111	63.1	76.6
	PDC041	7	716	600	116	83.8	101.7
	PDC019	7	668	558	110	83.5	101.4
	PDC032	7	322	269	53	83.5	101.4
	C037	8	389	331	58	85.1	103.3
	118375	5	339	232	107	68.4	83.1
	118089	7	276	274	2	99.3	120.5
	118467	5	354	290	64	81.9	99.4
	122016	7	273	243	30	89.0	108.0
PD	PD154	3	52	8	44	15.4	18.7
	PD427	4	51	35	16	68.6	83.3
	PD069	5	57	27	30	47.4	57.5
	PD110	5	38	14	24	36.8	44.7
	PD137	5	33	14	19	42.4	51.5
	PD506	5	72	40	32	55.6	67.4
	PD054	6	203	118	85	58.1	70.5
	PD141	6	65	27	38	41.5	50.4
	PD062	7	38	12	26	31.6	38.3
	PD161	7	100	44	56	44.0	53.4
	PD199	8	21	16	5	76.2	92.5
	Mean (control)	5.7	350.2	284.6	62.3	82.4	100.0
	Mean (PD & PDD)	6.0	66.4	32.3	34.1	47.1	57.1
	SEM (control)	0.4	57.0	49.9	11.4	6.1	7.4
	SEM (PD & PDD)	0.4	23.2	14.0	9.5	5.1	6.2

Supplemental Table 3. TH and ALDH1A1 in SNc of control and age-matched PD cases with immunofluorescence labeling.
